# Implantation of a Continuous-Flow Left Ventricular Assist Device During Cardiopulmonary Bypass Is Associated with a Significant and Transient Acute Thromboinflammatory Response

**DOI:** 10.3390/ijms27104594

**Published:** 2026-05-20

**Authors:** Alexandra Gerogianni, Gro Sørensen, Tom Nilsen Hoel, Karin E. McAdam, Camilla Schjalm, Einar Gude, Dick J. Sjöström, Carola Henriksson, Camilla Mohlin, Andreas Barratt-Due, Arnt Fiane, Tom E. Mollnes, Per H. Nilsson

**Affiliations:** 1 Linnaeus Centre for Biomaterials Chemistry, Linnaeus University, SE-39182 Kalmar, Sweden; 2 Department of Chemistry and Biomedicine, Linnaeus University, SE-39182 Kalmar, Sweden; 3 Department of Cardiothoracic Surgery, Oslo University Hospital, Rikshospitalet, NO-0372 Oslo, Norway; 4 Department of Immunology, Oslo University Hospital, University of Oslo, NO-0372 Oslo, Norway; 5 Department of Cardiology, Oslo University Hospital, Rikshospitalet, NO-0372 Oslo, Norway; 6 Department of Medical Biochemistry, Oslo University Hospital, Rikshospitalet, NO-0372 Oslo, Norway; 7 Research Laboratory, Nordland Hospital, NO-8092 Bodo, Norway

**Keywords:** left ventricular assist device, complement activation, thromboinflammation, inflammation, cytokines

## Abstract

Left ventricular assist device (LVAD) implantation is a life-saving therapy for end-stage heart failure but may compromise immune integrity. Mechanical shear stress and surface-induced innate immune activation can trigger bleeding and thromboembolic complications. While thrombotic mechanisms are well characterized, the associated inflammatory response remains poorly studied. We investigated thromboinflammation in patients with terminal heart failure (*n* = 8) implanted with the HeartWare ventricular assist device. Blood samples were collected before and immediately after implantation, daily for five days, and three months post-implantation. Ten age- and sex-matched healthy individuals served as controls. Samples were analyzed for a broad panel of thromboinflammatory and cell damage biomarkers. Twenty-eight of 43 biomarkers were significantly elevated (*p* < 0.05) at patient baseline compared with controls, indicating a pre-existing low-grade inflammatory state prior to LVAD implantation. Complement activation products increased markedly immediately after implantation—C3bc, C3bBbP, and the terminal complement complex C5b-9 rose 2.8-, 8.9-, and 6.6-fold, respectively, compared with baseline (*p* < 0.0001), but returned toward baseline within 24 h. A similar transient increase was observed for TNF, IL-6, IL-8, IL-10, IFN-γ, MMP-8, MMP-9, tissue factor, and prothrombin fragment 1.2 (*p* < 0.05). LVAD implantation with cardiopulmonary bypass induces a strong but transient immune response, including robust complement activation. Targeting upstream complement pathways may help attenuate downstream thromboinflammatory processes during the acute post-implantation period.

## 1. Introduction

Heart failure affects millions of patients worldwide [1]. Despite improvements in treatment, cardiac function eventually deteriorates [2], with impairment of ventricular function and blood circulation [3], leading to terminal heart failure [2]. This complex syndrome is associated with high rates of hospitalization and mortality. Heart transplantation is the most effective treatment [4], but various factors such as organ shortage, age, comorbidities, or reluctance to accept donor organs disqualify many patients from orthotopic heart transplantation [5]. Implantation of a left ventricular assist device (LVAD) has therefore been increasingly used as both bridge-to-transplantation and destination therapy [6] to improve survival and quality of life in patients with end-stage heart failure.

Exposure of implants to biological tissue is inevitably associated with a risk of host reactions to the artificial device. LVAD implantation is primarily associated with bleeding, pump thrombosis, and infection [7]. Bleeding is a frequent complication due to the combination of anticoagulant therapy and consumption of von Willebrand factor (vWF) larger-sized multimers, in some cases leading to acquired von Willebrand disease [7]. vWF is essential for platelet adhesion and aggregation at sites of blood vessel injury [8]. However, consumption of these multimers and impaired regulation caused by fluid shear stress may exacerbate bleeding [8]. Furthermore, LVAD can induce hemolysis [9], platelet activation and initiate the coagulation cascade via the extrinsic pathway through tissue factor (TF) expression following vascular injury. Coagulation may also be activated via the intrinsic pathway through the foreign surface-mediated activation of factor XII (FXII). Both mechanisms can lead to thrombus formation, including stroke and, in particular, pump thrombosis [10,11]. Thromboembolic events are significantly reduced with the newer HeartMate 3 device compared with HeartWare [12], demonstrating the importance of pump design and engineering.

LVAD implantation also entails a risk of inflammation through the activation of the host innate immune system. The surgical procedure itself, including cardiopulmonary bypass, in combination with exposure to the foreign surfaces of the LVAD, may trigger host immune reactions. The complement system is a major upstream part of innate immunity and is activated on surfaces lacking the capacity for active regulation. Complement activation may also occur secondary to tissue damage associated with cardiac surgery [13], including cardiopulmonary bypass [14], hemolysis [15,16], or thrombosis [17]. The complement system can be activated via three distinct pathways: the classical, lectin, and alternative pathways [18]. All three converge at the formation of enzymatic convertases that cleave C3 into C3a and C3b, and subsequently, C5 into C5a and C5b, leading to formation of the terminal C5b-9 complement complex (TCC) [19]. C3a and C5a are anaphylatoxins that activate immune cells by upregulating integrins involved in cell–cell interactions and by inducing the expression and release of multiple inflammatory mediators, including cytokines.

Previous LVAD research has primarily focused on clinical thrombotic events [20,21,22]. However, the inflammatory response during implantation and its connection to thrombosis as an important biological “crosstalk” may represent a potential opportunity for therapeutic targeting. In this study, we characterized the thromboinflammatory biomarker profile in patients undergoing implantation of the HeartWare LVAD, with a particular focus on complement activation and other inflammatory biomarkers.

## 2. Results

An extensive panel of biomarkers related to thromboinflammation and cell damage was analyzed in samples collected from eight patients undergoing LVAD implantation and ten age- and sex-matched healthy controls. Patient samples were obtained at eight time points: before implantation (baseline), immediately after implantation (Day 0), on five consecutive days post-implantation (Days 1–5), and at a three-month follow-up. Samples were missing for two patients at Day 0 and for two patients at the three-month follow-up. A descriptive heatmap summarizing relative changes in all analyzed biomarkers across the study period is presented in Supplementary Figure S1.

### 2.1. Hemolysis Markers

**Figure 1:**
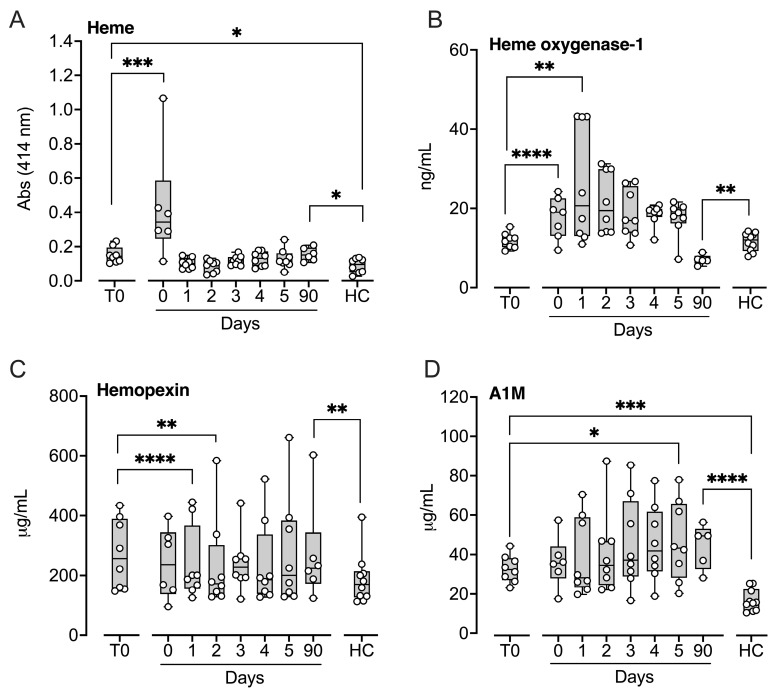
Hemolysis markers in patients subjected to LVAD implantation and healthy controls. Blood samples were collected from patients at baseline before LVAD implantation (T0), immediately after implantation (Day 0) and for the five consecutive days after implantation (Days 1–5), and at a three-month follow-up control (90 days). Age- and sex-matched healthy controls were sampled once. Plasma heme (**A**) was measured by spectrophotometric absorbance at 414 nm in EDTA plasma. Heme oxygenase-1 (**B**), hemopexin (**C**), and alpha-1-microglobulin (A1M) (**D**) were quantified in citrate plasma by ELISA. Results are presented in box and whisker plots showing all data points as symbols. Significant differences between before, i.e., time zero (T0), and after implantation were statistically determined using repeated measures ANOVA with Dunnett’s correction. Significant differences between controls and (i) patients at T0 and (ii) patients at three-month follow-up were statistically determined using ANOVA with Dunnett’s correction, * *p* < 0.05, ** *p* < 0.01, *** *p* < 0.001, **** *p* < 0.0001.

Four markers of hemolysis were assessed. Patients exhibited significantly elevated levels of heme and alpha-1-microglobulin (A1M) at baseline compared with healthy controls (*p* < 0.05 and *p* < 0.001, respectively), whereas no differences were observed for heme oxygenase-1 (HO-1) or hemopexin (Figure 1). Heme levels increased significantly immediately after implantation compared with baseline (*p* < 0.001) and remained elevated at the three-month follow-up (*p* < 0.05; Figure 1A). HO-1, the enzyme responsible for heme degradation, increased significantly immediately after implantation (*p* < 0.0001) and remained elevated at 24 h (*p* < 0.01) (Figure 1B), but was significantly lower than control levels at the three-month follow-up (*p* < 0.01). Hemopexin levels decreased significantly on Day 1 (*p* < 0.0001) and Day 2 (*p* < 0.01) compared with baseline (Figure 1C). In contrast, A1M levels increased significantly five days after implantation (*p* < 0.05; Figure 1D). Both hemopexin and A1M were significantly elevated at three months compared with controls (*p* < 0.01 and *p* < 0.0001, respectively).

### 2.2. Complement Activation Markers

**Figure 2:**
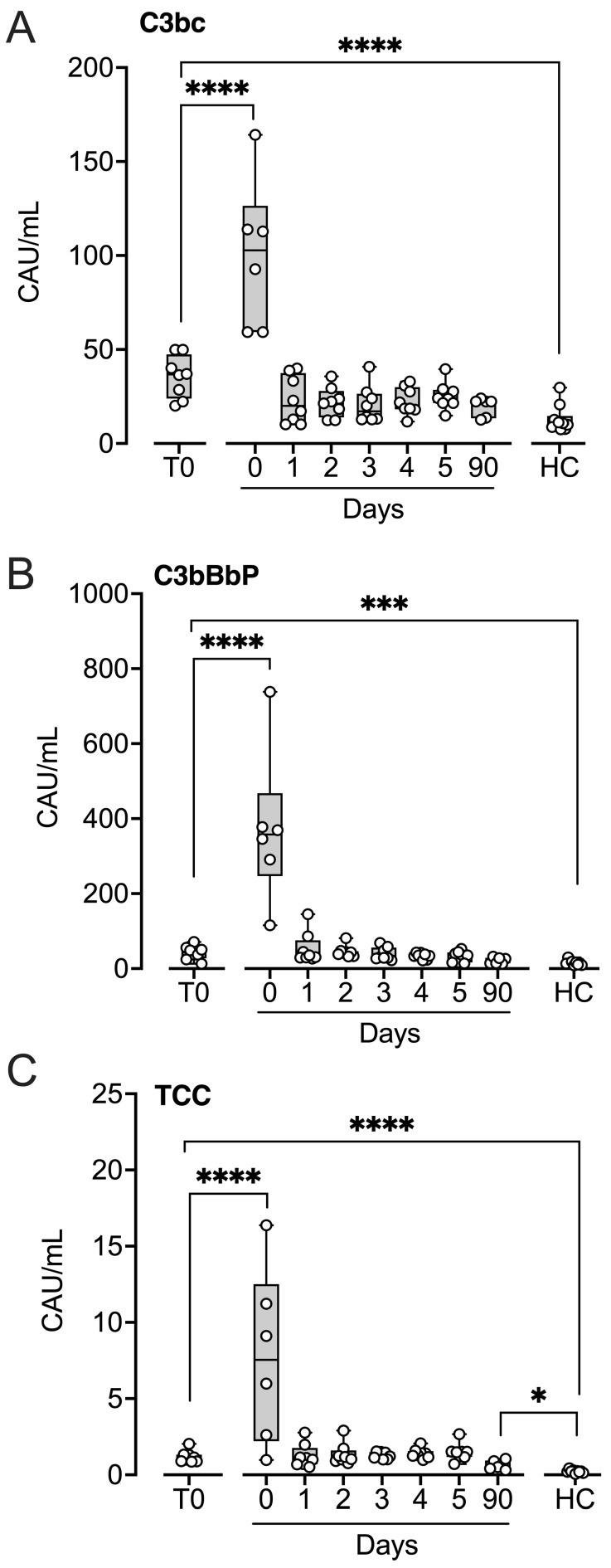
Complement activation markers in patients subjected to LVAD implantation and healthy controls. Blood samples were collected from patients at baseline before LVAD implantation (T0), immediately after implantation (Day 0) and for the five consecutive days after implantation (Days 1–5), and at a three-month follow-up control (90 days). Age- and sex-matched healthy controls were sampled once. Complement activation markers C3bc (**A**), C3bBbP (**B**), and terminal complement complex (TCC) (**C**) were quantified in EDTA plasma by ELISA, expressed as complement activating units per mL (CAU/mL). Results are presented in box and whisker plots showing all data points as symbols. Significant differences between baseline (T0) and after implantation were statistically determined using repeated measures ANOVA with Dunnett’s correction. Significant differences between controls and (i) patients at T0 and (ii) patients at three-month follow-up were statistically determined using ANOVA with Dunnett’s correction, * *p* < 0.05, *** *p* < 0.001, **** *p* < 0.0001.

All three complement activation markers—C3bc, C3bBbP, and TCC—were significantly elevated in patients at baseline compared with controls (all *p* < 0.001; Figure 2). Immediately after implantation, C3bc increased 2.8-fold (Figure 2A), C3bBbP increased 8.9-fold (Figure 2B), and TCC increased 6.6-fold (Figure 2C) (*p* < 0.001–*p* < 0.0001). Levels of all three markers returned to baseline within 24 h. At the three-month follow-up, only TCC remained significantly elevated compared with controls (*p* < 0.05).

### 2.3. Hemostatic Markers

**Figure 3:**
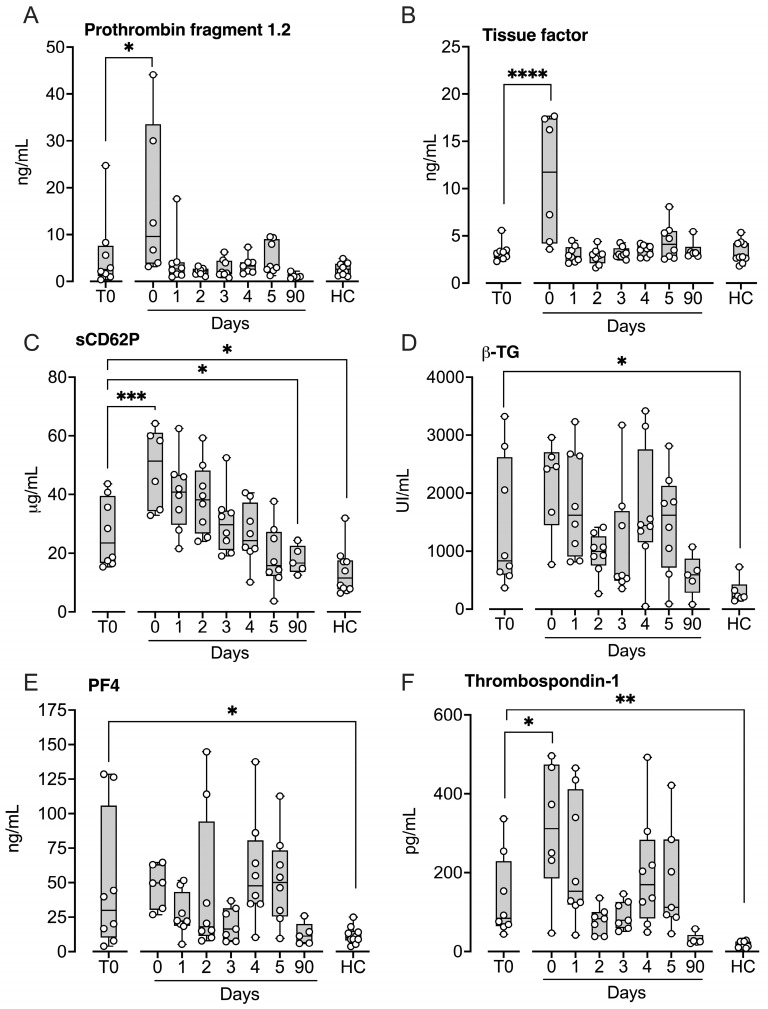
Hemostatic markers in patients subjected to LVAD implantation and healthy controls. Blood samples were collected from patients at baseline before LVAD implantation (T0), immediately after implantation (Day 0) and for the five consecutive days after implantation (Days 1–5), and at a three-month follow-up control (90 days). Age- and sex-matched healthy controls were sampled once. Prothrombin fragment 1.2 (**A**), microparticle tissue factor (**B**), soluble (s) CD62P (**C**), beta-thromboglobulin (β-TG) (**D**), platelet factor 4 (PF4) (**E**), and thrombospondin-1 (**F**) were quantified in EDTA (**A**), citrate (**B**) or CTAD plasma (**C**–**F**) by ELISA. Results are presented in box and whisker plots showing all data points as symbols. Significant differences between baseline (T0) and after implantation were statistically determined using repeated measures ANOVA with Dunnett’s correction. Significant differences between controls and (i) patients at T0 and (ii) patients at three-month follow-up were statistically determined using ANOVA with Dunnett’s correction, * *p* < 0.05, ** *p* < 0.01, *** *p* < 0.001, **** *p* < 0.0001.

Neither tissue factor (TF), reflecting activation of the extrinsic coagulation pathway, nor prothrombin fragment 1.2 (PF 1.2) differed significantly from controls immediately after implantation or at three months (Figure 3). PF 1.2 (Figure 3A) and TF (Figure 3B) increased significantly after implantation (*p* < 0.05 and *p* < 0.0001, respectively) and subsequently returned to baseline levels. Among platelet activation markers, soluble P-selectin (sCD62P) was significantly elevated at baseline compared with controls (*p* < 0.05; Figure 3C), increased further after implantation (*p* < 0.001), and then decreased by Day 1, reaching levels significantly lower than baseline at three months (*p* < 0.05). β-thromboglobulin (β-TG) (Figure 3D), platelet factor 4 (PF4) (Figure 3E), and thrombospondin-1 (TSP-1) (Figure 3F) were all significantly increased at baseline (*p* < 0.05–*p* < 0.01), but only TSP-1 increased further following implantation.

### 2.4. Cytokines, Including Interleukins, Chemokines, and Growth Factors

**Figure 4:**
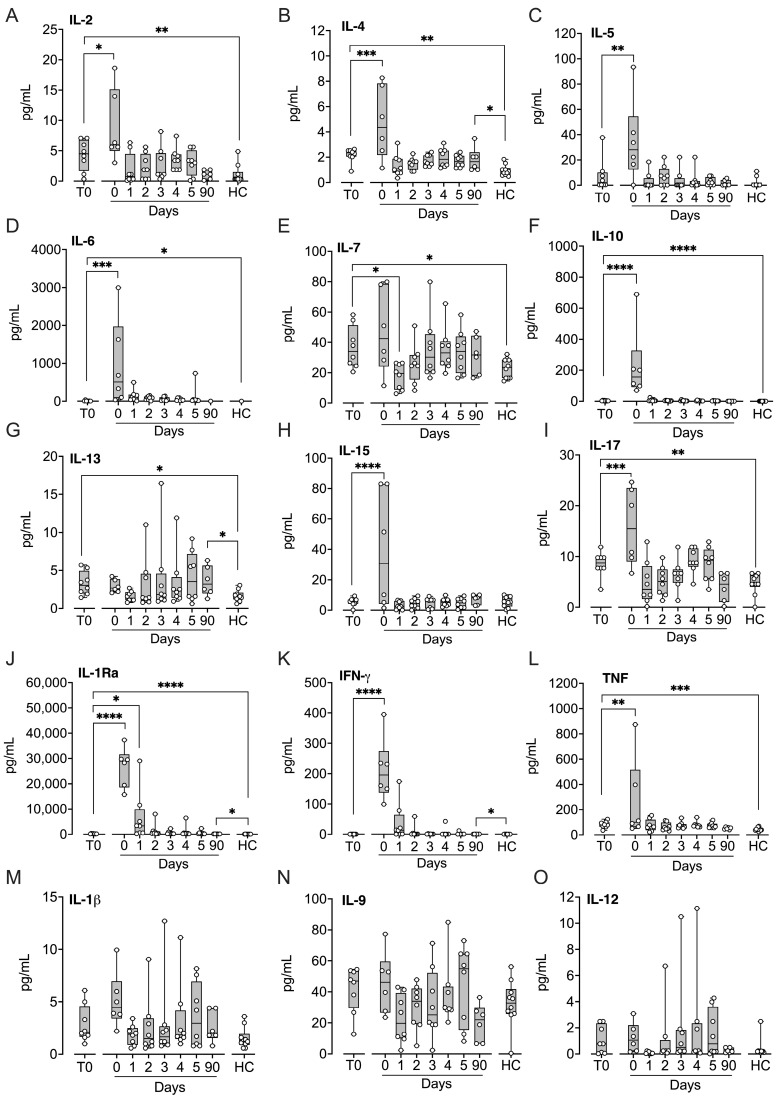
Cytokines, including interleukins, IFN-γ, and TNF, in patients subjected to LVAD implantation and healthy controls. Blood samples were collected from patients at baseline before LVAD implantation (T0), immediately after implantation (Day 0) and for the five consecutive days after implantation (Days 1–5), and at a three-month follow-up control (90 days). Age- and sex-matched healthy controls were sampled once. IL-2 (**A**), IL-4 (**B**), IL-5 (**C**), IL-6 (**D**), IL-7 (**E**), IL-10 (**F**), IL-13 (**G**), IL-15 (**H**), IL-17 (**I**), IL-1Ra (**J**), IFN-γ (**K**), TNF (**L**), IL-1β (**M**), IL-9 (**N**), and IL-12 (**O**) were quantified in EDTA plasma by Luminex MagPix system. Results are presented in box and whisker plots showing all data points as symbols. Significant differences between baseline (T0) and after implantation were statistically determined using repeated measures ANOVA with Dunnett’s correction. Significant differences between controls and (i) patients at T0 and (ii) patients at three-month follow-up were statistically determined using ANOVA with Dunnett’s correction, * *p* < 0.05, ** *p* < 0.01, *** *p* < 0.001, **** *p* < 0.0001.

Of the 15 cytokines analyzed, nine were significantly elevated at baseline compared with controls: interleukin (IL)-2 (*p* < 0.01), IL-4 (*p* < 0.01), IL-6 (*p* < 0.05), IL-7 (*p* < 0.05), IL-10 (*p* < 0.0001), IL-13 (*p* < 0.05), IL-17 (*p* < 0.01), IL-1 receptor antagonist (IL-1Ra) (*p* < 0.0001), and tumor necrosis factor (TNF) (*p* < 0.001) (Figure 4). Following implantation, IL-2, IL-4, IL-5, IL-6, IL-10, IL-15, IL-17, IL-1Ra, interferon-γ (IFN-γ), and TNF all increased significantly (*p* < 0.05–*p* < 0.0001), whereas IL-7 decreased at Day 1 (*p* < 0.05). At three months, IL-4, IL-13, IL-1Ra, and IFN-γ remained significantly elevated compared with controls (all *p* < 0.05). IL-1β, IL-9, and IL-12 did not differ significantly across any of the comparisons.

**Figure 5:**
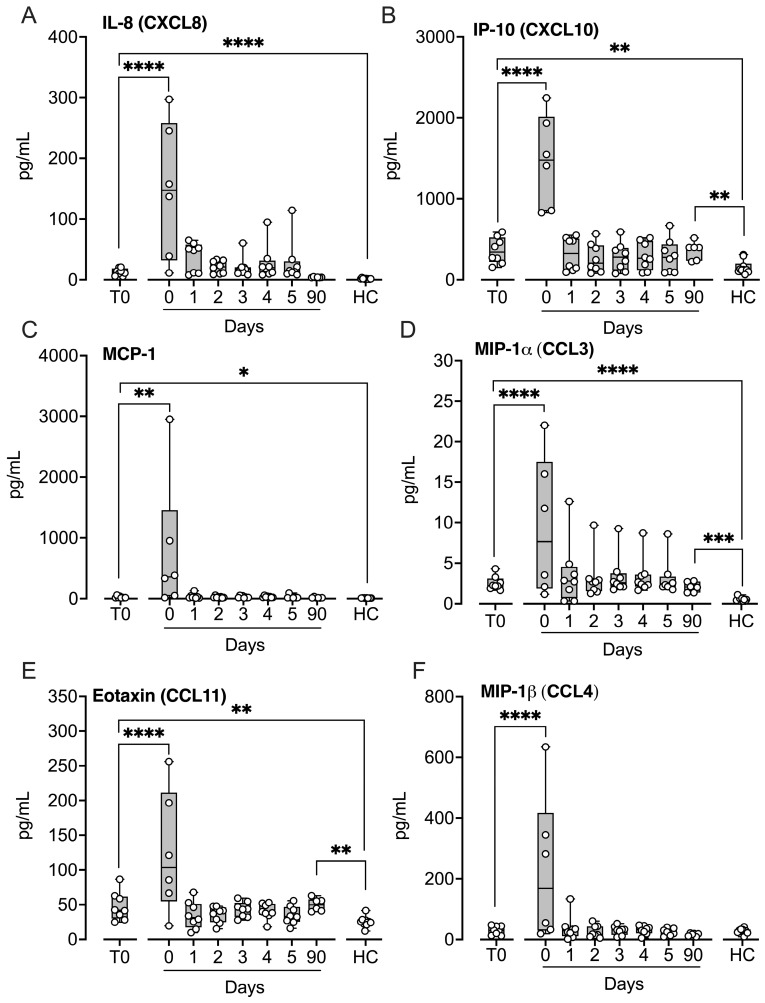
Chemokines in patients subjected to LVAD implantation and healthy controls. Blood samples were collected from patients at baseline before LVAD implantation (T0), immediately after implantation (Day 0) and for the five consecutive days after implantation (Days 1–5), and at a three-month follow-up control (90 days). Age- and sex-matched healthy controls were sampled once. IL-8 (**A**), IP-10 (**B**), MCP-1 (**C**), MIP-1α (**D**), eotaxin (**E**), and MIP-1β (**F**) were quantified in EDTA plasma by Luminex MagPix system. Results are presented in box and whisker plots showing all data points as symbols. Significant differences between baseline (T0) and after implantation were statistically determined using repeated measures ANOVA with Dunnett’s correction. Significant differences between controls and (i) patients at T0 and (ii) patients at three-month follow-up were statistically determined using ANOVA with Dunnett’s correction, * *p* < 0.05, ** *p* < 0.01, *** *p* < 0.001, **** *p* < 0.0001.

Of the six chemokines analyzed, IL-8, interferon gamma-induced protein 10 (IP-10), monocyte chemoattractant protein-1 (MCP-1), macrophage inflammatory protein (MIP)-1α, and eotaxin, but not MIP-1β, were significantly elevated at baseline compared with controls (*p* < 0.05–*p* < 0.0001; Figure 5). All six chemokines increased markedly immediately after implantation (*p* < 0.01–*p* < 0.0001) and returned to baseline within 24 h. IP-10, MIP-1α, and eotaxin remained significantly elevated at the three-month follow-up (*p* < 0.01–*p* < 0.001).

**Figure 6:**
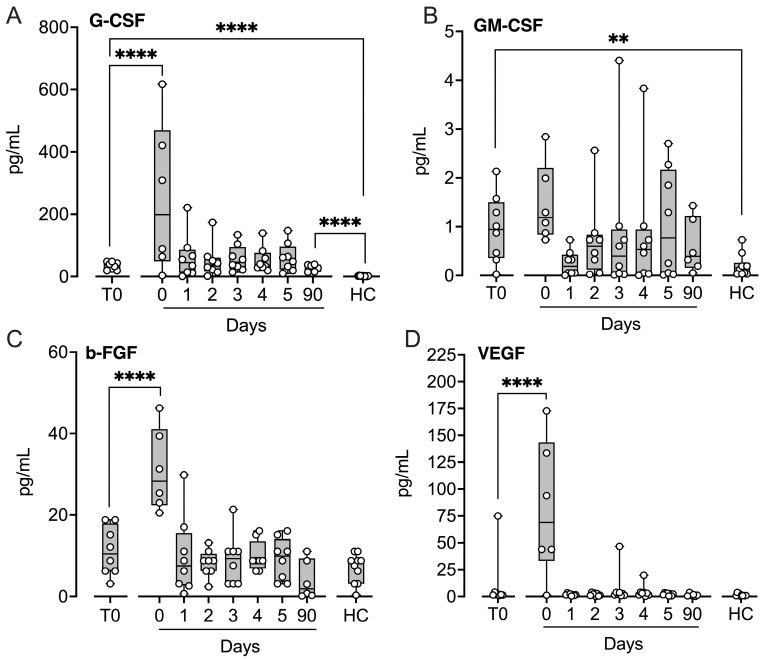
Growth factors in patients subjected to LVAD implantation and healthy controls. Blood samples were collected from patients at baseline before LVAD implantation (T0), immediately after implantation (Day 0) and for the five consecutive days after implantation (Days 1–5), and at a three-month follow-up control (90 days). Age- and sex-matched healthy controls were sampled once. G-CSF (**A**), GM-CSF (**B**), basic fibroblast growth factor (b-FGF) (**C**), and vascular endothelial growth factor (VEGF) (**D**) were quantified in EDTA plasma by the Luminex MagPix^®^ system. Results are presented in box and whisker plots showing all data points as symbols. Significant differences between baseline (T0) and after implantation were statistically determined using repeated measures ANOVA with Dunnett’s correction. Significant differences between controls and (i) patients at T0 and (ii) patients at three-month follow-up were statistically determined using ANOVA with Dunnett’s correction, ** *p* < 0.01, **** *p* < 0.0001.

Four growth factors were assessed. Granulocyte colony-stimulating factor G-CSF (Figure 6A) and granulocyte–macrophage colony-stimulating factor (GM-CSF) (Figure 6B) were significantly increased at baseline compared with controls (*p* < 0.01–*p* < 0.0001), whereas basic fibroblast growth factor (bFGF) (Figure 6C) and vascular endothelial growth factor (VEGF) (Figure 6D) were not. All growth factors, except GM-CSF, increased significantly immediately after implantation (*p* < 0.0001). All returned to baseline within 24 h, except G-CSF, which remained elevated at three months (*p* < 0.0001).

### 2.5. MMPs and TIMP-1

**Figure 7:**
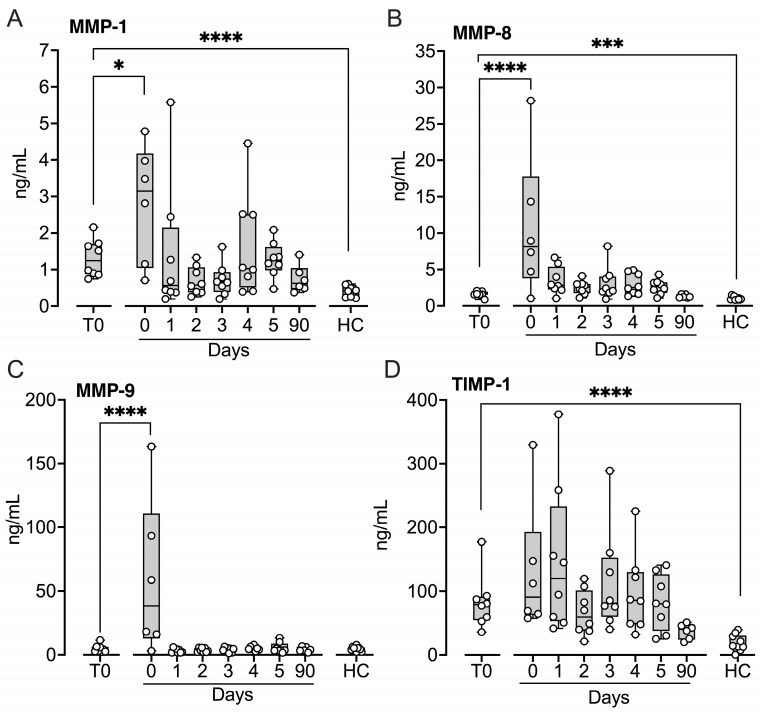
Matrix metalloproteinases in patients subjected to LVAD implantation and healthy controls. Blood samples were collected from patients at baseline before LVAD implantation (T0), immediately after implantation (Day 0) and for the five consecutive days after implantation (Days 1–5), and at a three-month follow-up control (90 days). Age- and sex-matched healthy controls were sampled once. Matrix metalloproteinase (MMP)-1 (**A**), MMP-8 (**B**), and MMP-9 (**C**) were quantified in EDTA plasma by Luminex MagPix system, and tissue inhibitor of metalloproteinases-1 (TIMP-1) (**D**) was quantified in EDTA plasma by ELISA. Results are presented in box and whisker plots showing all data points as symbols. Significant differences between baseline and after implantation were statistically determined using repeated measures ANOVA with Dunnett’s correction. Significant differences between controls and (i) patients at T0 and (ii) patients at three-month follow-up were statistically determined using ANOVA with Dunnett’s correction, * *p* < 0.05, *** *p* < 0.001, **** *p* < 0.0001.

Matrix metalloproteinases (MMP)-1 (Figure 7A), MMP-8 (Figure 7B), but not MMP-9 (Figure 7C), were significantly increased at baseline compared with controls (*p* < 0.001–*p* < 0.0001). The inhibitor of MMPs, tissue inhibitor of metalloproteinases-1 (TIMP-1) (Figure 7D), was also increased at baseline compared with controls (*p* < 0.0001). MMP-1, MMP-8, and MMP-9 increased significantly immediately after implantation (*p* < 0.05–*p* < 0.0001) and returned to baseline on Day 1.

### 2.6. CRP and Hematological Parameters

**Figure 8:**
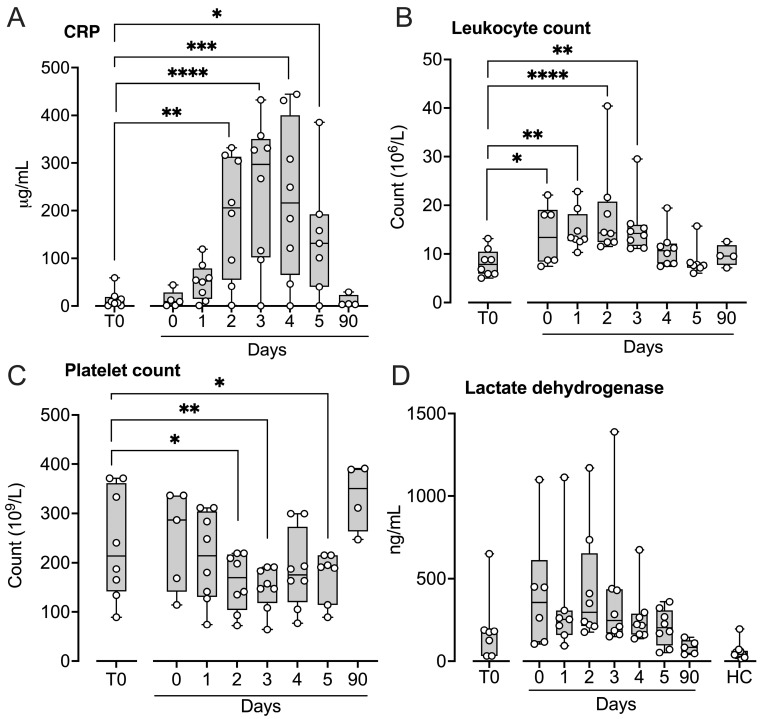
C-reactive protein and hematological parameters in patients subjected to LVAD implantation. Blood samples were collected from patients at baseline before LVAD implantation (T0), immediately after implantation (Day 0) and for the five consecutive days after implantation (Days 1–5), and at a three-month follow-up control (90 days). C-reactive protein (CRP) (**A**), leukocyte- (**B**) and platelet counts (**C**), and lactate dehydrogenase (**D**) were determined. Results are presented in box and whisker plots showing all data points as symbols. Significant differences between baseline and after implantation were statistically determined using repeated measures ANOVA with Dunnett’s correction. Significant differences between controls and (i) T0 at baseline and (ii) patients at three-month follow-up were statistically determined using ANOVA with Dunnett’s correction, * *p* < 0.05, ** *p* < 0.01, *** *p* < 0.001, **** *p* < 0.0001.

C-reactive protein (CRP), leukocyte counts, and platelet counts were assessed only in patient samples (Figure 8). CRP increased from a baseline mean of 14 ± 19 µg/mL to a peak of 240 ± 151 µg/mL on Day 3 (Figure 8A), with statistically significant increases from Day 2 through Day 5 (*p* < 0.05–*p* < 0.0001). Leukocyte counts rose significantly immediately after implantation (*p* < 0.05), peaked on Day 2 (*p* < 0.0001), and remained elevated on Day 3 (*p* < 0.01) (Figure 8B). Platelet counts decreased significantly on Day 2 (*p* < 0.05) and remained reduced on Days 3 and 5 (*p* < 0.01 and *p* < 0.05, respectively) (Figure 8C). Lactate dehydrogenase (LDH), used as a marker of cell damage, did not change significantly throughout the study period (Figure 8D).

## 3. Discussion

LVAD implantation involves cardiac surgery with cardiopulmonary bypass, exposure to artificial surfaces, and anticoagulant therapy, and thus imposes substantial acute stress on the host immune system. In this study, we quantified a broad panel of biomarkers to characterize the thromboinflammatory response during the first five days after implantation of the HeartWare LVAD. The majority of inflammatory markers were markedly elevated immediately after implantation, including activation of the complement and hemostatic systems, together with a pronounced cytokine response and release of metalloproteinases. However, most markers returned to baseline levels within 24 h. This temporal pattern suggests that cardiopulmonary bypass is the primary driver of the acute response and supports an overall acceptable biocompatibility of the LVAD device in the postoperative phase.

The rapid but transient thromboinflammatory response observed here is consistent with previous reports on cardiac surgery involving cardiopulmonary bypass alone [13,14,23]. This suggests that the procedure itself, rather than the device, is the major driver of the acute response. Corry et al. described the temporal profiles of IL-6, IL-8, and C3a in patients undergoing implantation of the HeartMate II LVAD, demonstrating immediate postoperative increases that were primarily attributed to the bypass procedure rather than the device itself [24]. In contrast to our findings, sustained elevations of IL-6 and IL-8 at 24 h post-implantation were reported, which were attributed to device-related effects. In our cohort, only IL-1Ra remained significantly elevated 24 h after implantation, suggesting a more transient inflammatory response. However, our study design does not allow separation of effects caused by cardiopulmonary bypass from those potentially attributable to the LVAD itself due to the absence of a cardiopulmonary bypass-only control group.

Although increased levels of IL-8, IL-10, and IL-1Ra in the early postoperative phase (4 h to 7 days) have been associated with multiple organ failure in LVAD recipients [25], inflammation per se is generally not considered the primary driver of LVAD-related complications. Instead, clinical complications are more strongly linked to disturbances in hemostasis, including both thrombotic and bleeding events, which complicate anticoagulant and antiplatelet management. Complement activation products such as C5a and pro-inflammatory cytokines including TNF can induce tissue factor expression, providing a mechanistic link between inflammation and coagulation [26,27,28]. This interaction highlights inflammation as a potentially modifiable upstream contributor to thrombotic risk.

The clinical relevance of complement activation in thrombotic disease is exemplified by paroxysmal nocturnal hemoglobinuria, in which uncontrolled complement activation leads to hemolysis and severe thrombosis. Treatment with complement inhibitors that block C5 not only prevents hemolysis but also markedly reduces thrombotic complications [29]. Similarly, complement inhibition has shown beneficial effects in the setting of extracorporeal circulation [30] and cardiac surgery [31]. Both pexelizumab, a single-chain anti-C5 antibody fragment, and TP10, a soluble complement receptor 1 derivative targeting C3b in the C3 and C5 convertases, have demonstrated safety and attenuation of complement activation during cardiopulmonary bypass [32,33]. With several novel complement inhibitors now available for clinical use [34], targeted modulation of complement activation may represent a promising strategy to reduce thromboinflammatory complications during LVAD implantation.

Notably, most biomarkers were already elevated at baseline compared with healthy controls, indicating a pre-existing inflammatory state in these patients. This likely reflects chronic heart failure-associated immune activation, driven by tissue hypoxia and impaired microcirculation [35]. Such pre-existing inflammation may either predispose patients to exaggerated inflammatory and thrombotic responses during device implantation or lead to biologically driven ceiling effects, limiting the magnitude of observable relative increases for certain markers post-implantation.

The HeartWare LVAD was withdrawn from the market in 2021 due to safety concerns related to thromboembolic and neurological complications [36], which limits the direct clinical applicability of the present findings to current LVAD practice. Newer devices, such as the HeartMate 3, differ substantially in pump design, flow characteristics, and hemocompatibility, and are associated with lower rates of thrombotic and neurological complications [37]. Therefore, extrapolation of the present results to contemporary devices should be made with caution. Nevertheless, the pronounced and transient activation of complement and inflammatory pathways observed here likely reflects upstream mechanisms related to cardiopulmonary bypass, surgical trauma, and blood–material interactions, which remain relevant across LVAD platforms.

This study has limitations. The cohort size was small (*n* = 8), which limits statistical power to detect moderate effect sizes and increases susceptibility to inter-individual variability and outliers. Consequently, effect size estimates and variability measures should be interpreted with caution and considered hypothesis-generating rather than definitive. In addition, missing samples at Day 0 and at the three-month follow-up in two patients further reduced the effective sample size at these time points. However, the dense longitudinal sampling and the consistent temporal patterns observed across multiple biomarkers, particularly the rapid and transient activation of complement and inflammatory pathways immediately after implantation, support the robustness of the main mechanistic conclusions. Second, the absence of a control group undergoing cardiopulmonary bypass without LVAD implantation precludes attribution of the acute thromboinflammatory response specifically to the device. Third, patients received pre- and postoperative medications, including anticoagulants, vasoactive agents, and antiarrhythmics, in clinically individualized regimens. These treatments may have influenced hemostatic and platelet-related biomarkers, such as prothrombin fragment 1.2, tissue factor, and soluble CD62P.

In conclusion, LVAD implantation during cardiopulmonary bypass is associated with a pronounced but transient thromboinflammatory response, dominated by early complement activation and cytokine release. Although inflammation does not appear to be the primary driver of adverse outcomes, it may amplify thrombotic risk through crosstalk with the coagulation system. Targeting upstream inflammatory pathways, particularly complement activation, may therefore represent a promising strategy to mitigate thromboinflammatory complications in the acute post-implantation period.

## 4. Materials and Methods

### 4.1. Study Design

Patients with advanced heart failure (*n* = 8), aged ≥ 18 years, who qualified for LVAD implantation between January 2020 and May 2021 were included in this study. Baseline clinical and demographic characteristics of the patient cohort are provided in Table 1. The HeartWare LVAD system (HeartWare, Medtronic, Minneapolis, MN, USA) was implanted according to standard surgical procedures, including normothermic cardiopulmonary bypass, as previously described [38]. Anticoagulation therapy was individualized for each patient. Warfarin and aspirin were administered postoperatively and continued throughout the study period. No thromboembolic events or major bleeding complications were observed postoperatively. No cases of antithrombotic drug intolerance were registered during clinical follow-up. In this observational study, blood samples were obtained before implantation (baseline), 2–4 h after implantation, daily for the following five consecutive days, and at a follow-up visit three months after implantation. Volunteers, without known cardiovascular or inflammatory disease, were included as a control group for baseline comparisons. Controls were age- and sex-matched to the patients, and blood samples were collected once.

### 4.2. Sample Collection

During hospitalization, blood samples were initially collected from the radial artery via an indwelling arterial catheter and subsequently obtained by venipuncture after catheter removal. At each time point, samples were collected in vacutainer tubes (BD Vacutainer Systems, Franklin Lakes, NJ, USA) containing either EDTA (ethylenediaminetetraacetic acid), CTAD (citrate–theophylline–adenosine–dipyridamole), or citrate as anticoagulants. Blood samples were immediately processed for hematological analyses or prepared for storage. EDTA plasma was obtained by centrifugation (Eppendorf 5424R, Eppendorf, Hamburg, Germany) at 3000× *g* for 15 min. CTAD- and citrate-anticoagulated blood was centrifuged at 2500× *g* for 15 min at 25 °C; citrate samples were centrifuged a second time under identical conditions. All plasma samples were aliquoted and stored at −70 °C within one hour of collection.

### 4.3. Hemolysis Markers

EDTA plasma heme levels were assessed by recording absorbance spectra between 350 and 700 nm using a NanoDrop™ spectrophotometer (Thermo Scientific, Stockholm, Sweden). The absorbance at 414 nm, corresponding to the heme Soret peak, was used for analysis. Hemopexin was measured by ELISA, as previously described [16]. HO-1, LDH, and A1M were quantified in citrate plasma using ELISA kits from Abcam (Cambridge, UK) according to the manufacturer’s instructions.

### 4.4. Complement Activation Markers

EDTA plasma was sampled and analyzed for complement activation markers according to the recommended routines [39]. C3bc, C3bBbP and soluble TCC were analyzed using enzyme-linked immunosorbent assays (ELISAs), as previously described [40].

### 4.5. Hemostatic Markers

PF 1.2 was measured in EDTA plasma using a commercial ELISA (Siemens Healthcare Diagnostics, Marburg, Germany) according to the manufacturer’s instructions. TF was measured in citrate anticoagulated plasma using the Zymuphen MP-TF immunoassay (Hyphen BioMed, Neuville Sur Oise, France) according to the manufacturer’s instructions. Soluble platelet activation markers, sCD62P, TSP-1, PF4, and β-TG, were measured in CTAD plasma. sCD62P, TSP-1, and PF4 were quantified using ELISA DuoSet kits from R&D Systems, Minneapolis, MN, USA, and β-TG was measured using an ELISA from Diagnostica Stago (Parsippany, NJ, USA). All assays were performed according to the manufacturers’ protocols.

### 4.6. Cytokines, Including Interleukins, Chemokines, and Growth Factors

Cytokines were measured in EDTA plasma using a Bio-Plex human 27-plex assay (Bio-Rad Laboratories, Hercules, CA, USA), including IL-1β, IL-1Ra, IL-2, IL-4, IL-5, IL-6, IL-7, IL-8 (CXCL8), IL-9, IL-10, IL-12, IL-13, IL-15, IL-17, MCP-1/CCL2, MIP-1α/CCL3, MIP-1β (CCL4), eotaxin (CCL11), IP-10/CXCL10, bFGF, G-CSF, GM-CSF, IFN-γ, TNF, and VEGF. Measurements were performed on a Luminex MagPix system according to the manufacturer’s instructions.

### 4.7. MMPs and TIMP-1

MMP-1, MMP-8, and MMP-9 were quantified in EDTA plasma using a multiplex assay (R&D Systems, Minneapolis, MN, USA) on the Luminex MagPix platform. TIMP-1 was measured in EDTA plasma using ELISA DuoSet kit (R&D Systems, Minneapolis, MN, USA) according to the manufacturer’s protocols.

### 4.8. C-Reactive Protein and Hematological Parameters

CRP was measured using a particle-enhanced turbidimetric immunoassay on a Modular P800 analyzer (Roche, Basel, Switzerland). Platelet and leukocyte counts were determined using an automated hematology analyzer (Cell-Dyn Sapphire, Abbott, IL, USA).

### 4.9. Statistics

Differences between baseline (pre-implantation) and post-implantation time points were analyzed using repeated-measures one-way ANOVA followed by Dunnett’s post hoc test. Comparisons between patients at baseline and healthy controls, as well as between patients at the three-month follow-up and healthy controls, were performed using ANOVA with Dunnett’s correction. A *p*-value < 0.05 was considered statistically significant. Statistical analyses and graphical representations were performed using GraphPad Prism version 9.5.0 (GraphPad Software, San Diego, CA, USA).

## Figures and Tables

**Table 1:** Baseline patient characteristics.

Number of patients	8
Sex, *n* (male/female)	7/1
Age, years	Median 55 (range 42–67)
NYHA functional class	III: 1; IV: 7
Body weight, kg	Median 83 (range 52–114)
Body mass index (BMI)	Median 25.4 (range 19.8–34.0)
Pre-implantation ejection fraction, %	Median 29 (range 27–32) ^a^
Heart failure etiology:	
- Cardiomyopathy, *n*	4
- Ischemic heart disease, *n*	4
Renal insufficiency, *n*	3
Diabetes, *n*	3
Pre-operative mechanical circulatory support, *n*	Yes: 6; No: 2

^a^ Ejection fraction available in 6/8 patients.

## Data Availability

The original contributions presented in this study are included in the article/Supplementary Materials. Further inquiries can be directed to the corresponding author.

## Supplementary Materials

The following supporting information can be downloaded at: https://www.mdpi.com/article/10.3390/ijms27104594/s1.

## Abbreviations

The following abbreviations are used in this manuscript:

A1M	Alpha-1-microglobulin
bFGF	Basic fibroblast growth factor
β-TG	Beta-thromboglobulin
CRP	C-reactive protein
CTAD	Citrate–theophylline–adenosine–dipyridamole
ELISA	Enzyme-linked immunosorbent assay
F	Coagulation factor
G-CSF	Granulocyte colony-stimulating factor
GM-CSF	Granulocyte–macrophage colony-stimulating factor
HO-1	Heme oxygenase-1
IFN-γ	Interferon-gamma
IL	Interleukin
IL-1Ra	Interleukin-1 receptor antagonist
IP-10	Interferon-gamma-induced protein 10
LVAD	Left ventricular assist device
MCP-1	Monocyte chemoattractant protein-1
MIP	Macrophage inflammatory protein
MMP	Matrix metalloproteinase
PF4	Platelet factor 4
sCD62P	Soluble CD62P (P-selectin)
TF	Tissue factor
TCC	Terminal complement complex (C5b-9)
TIMP-1	Tissue inhibitor of metalloproteinases-1
TNF	Tumor necrosis factor
TSP-1	Thrombospondin-1
VEGF	Vascular endothelial growth factor
vWF	von Willebrand factor

## References

[1] Radley G., Pieper I.L., Ali S., Bhatti F., Thornton C.A. (2018). The Inflammatory Response to Ventricular Assist Devices. Front. Immunol..

[2] Metra M., Dinatolo E., Dasseni N. (2019). The New Heart Failure Association Definition of Advanced Heart Failure. Card. Fail. Rev..

[3] Yancy C.W., Jessup M., Bozkurt B., Butler J., Casey D.E., Drazner M.H., Fonarow G.C., Geraci S.A., Horwich T., Januzzi J.L. (2013). 2013 ACCF/AHA guideline for the management of heart failure: Executive summary: A report of the American College of Cardiology Foundation/American Heart Association Task Force on practice guidelines. Circulation.

[4] Westerdahl D.E., Kobashigawa J.A., Brown D.L. (2019). 48—Heart Transplantation for Advanced Heart Failure. Cardiac Intensive Care.

[5] Dunlay S.M., Strand J.J., Wordingham S.E., Stulak J.M., Luckhardt A.J., Swetz K.M. (2016). Dying with a Left Ventricular Assist Device as Destination Therapy. Circ. Heart Fail..

[6] Patel S., Nicholson L., Cassidy C.J., Wong K.Y. (2016). Left ventricular assist device: A bridge to transplant or destination therapy?. Postgrad. Med. J..

[7] Aissaoui N., Jouan J., Gourjault M., Diebold B., Ortuno S., Hamdan A., Latremouille C., Pirracchio R., Morshuis M. (2018). Understanding Left Ventricular Assist Devices. Blood Purif..

[8] Jilma-Stohlawetz P., Quehenberger P., Schima H., Stoiber M., Knobl P., Steinlechner B., Felli A., Jilma B. (2016). Acquired von Willebrand factor deficiency caused by LVAD is ADAMTS-13 and platelet dependent. Thromb. Res..

[9] Heilmann C., Geisen U., Benk C., Berchtold-Herz M., Trummer G., Schlensak C., Zieger B., Beyersdorf F. (2009). Haemolysis in patients with ventricular assist devices: Major differences between systems. Eur. J. Cardiothorac. Surg..

[10] Walenga J.M., Torres T.A., Jeske W.P., Schwartz J., Escalante V., Newman J.D., Bakhos M. (2020). Protein C Pathway, Inflammation, and Pump Thrombosis in Patients with Left Ventricular Assist Devices. Clin. Appl. Thromb. Hemost..

[11] Boyle A.J., Russell S.D., Teuteberg J.J., Slaughter M.S., Moazami N., Pagani F.D., Frazier O.H., Heatley G., Farrar D.J., John R. (2009). Low thromboembolism and pump thrombosis with the HeartMate II left ventricular assist device: Analysis of outpatient anti-coagulation. J. Heart Lung Transplant..

[12] Francica A., Loforte A., Attisani M., Maiani M., Iacovoni A., Nisi T., Comisso M., Terzi A., De Bonis M., Vendramin I. (2023). Five-Year Outcome After Continuous Flow LVAD with Full-Magnetic (HeartMate 3) Versus Hybrid Levitation System (HeartWare): A Propensity-Score Matched Study from an All-Comers Multicentre Registry. Transpl. Int..

[13] Gu Y.J., Mariani M.A., Boonstra P.W., Grandjean J.G., van Oeveren W. (1999). Complement activation in coronary artery bypass grafting patients without cardiopulmonary bypass: The role of tissue injury by surgical incision. Chest.

[14] Hoel T.N., Videm V., Mollnes T.E., Saatvedt K., Brosstad F., Fiane A.E., Fosse E., Svennevig J.L. (2007). Off-pump cardiac surgery abolishes complement activation. Perfusion.

[15] Gerogianni A., Dimitrov J.D., Zarantonello A., Poillerat V., Chonat S., Sandholm K., McAdam K.E., Ekdahl K.N., Mollnes T.E., Mohlin C. (2022). Heme Interferes with Complement Factor I-Dependent Regulation by Enhancing Alternative Pathway Activation. Front. Immunol..

[16] Thomas A.M., Gerogianni A., McAdam M.B., Floisand Y., Lau C., Espevik T., Nilsson P.H., Mollnes T.E., Barratt-Due A. (2019). Complement Component C5 and TLR Molecule CD14 Mediate Heme-Induced Thromboinflammation in Human Blood. J. Immunol..

[17] Wiegner R., Chakraborty S., Huber-Lang M. (2016). Complement-coagulation crosstalk on cellular and artificial surfaces. Immunobiology.

[18] Andersson J., Ekdahl K.N., Lambris J.D., Nilsson B. (2005). Binding of C3 fragments on top of adsorbed plasma proteins during complement activation on a model biomaterial surface. Biomaterials.

[19] Dunkelberger J.R., Song W.C. (2010). Complement and its role in innate and adaptive immune responses. Cell Res..

[20] Grabska J., Schloglhofer T., Gross C., Maw M., Dimitrov K., Wiedemann D., Zimpfer D., Schima H., Moscato F. (2020). Early Detection of Pump Thrombosis in Patients with Left Ventricular Assist Device. ASAIO J..

[21] Fatullayev J., Samak M., Sabashnikov A., Zeriouh M., Rahmanian P.B., Choi Y.H., Schmack B., Kallenbach K., Ruhparwar A., Eghbalzadeh K. (2015). Continuous-Flow Left Ventricular Assist Device Thrombosis: A Danger Foreseen is a Danger Avoided. Med. Sci. Monit. Basic Res..

[22] Thenappan T., Anderson A.S., Jeevanadham V., Rich J.D., Shah A.P. (2013). Treatment of left ventricular assist device thrombosis with extended catheter-directed intraventricular thrombolytic therapy. Circ. Heart Fail..

[23] Bronicki R.A., Hall M. (2016). Cardiopulmonary Bypass-Induced Inflammatory Response: Pathophysiology and Treatment. Pediatr. Crit. Care Med..

[24] Corry D.C., DeLucia A., Zhu H., Radcliffe R.R., Brevetti G.R., El-Khatib H., Vance S.J., Moyer B.R., Cotts W.G., Richenbacher W.E. (1998). Time course of cytokine release and complement activation after implantation of the HeartMate left ventricular assist device. ASAIO J..

[25] Caruso R., Trunfio S., Milazzo F., Campolo J., De Maria R., Colombo T., Parolini M., Cannata A., Russo C., Paino R. (2010). Early expression of pro- and anti-inflammatory cytokines in left ventricular assist device recipients with multiple organ failure syndrome. ASAIO J..

[26] Cuccuini W., Poitevin S., Poitevin G., Dignat-George F., Cornillet-Lefebvre P., Sabatier F., Nguyen P. (2010). Tissue factor up-regulation in proinflammatory conditions confers thrombin generation capacity to endothelial colony-forming cells without influencing non-coagulant properties in vitro. J. Thromb. Haemost..

[27] Ward P.A. (2010). Role of C5 activation products in sepsis. Sci. World J..

[28] Ritis K., Doumas M., Mastellos D., Micheli A., Giaglis S., Magotti P., Rafail S., Kartalis G., Sideras P., Lambris J.D. (2006). A novel C5a receptor-tissue factor cross-talk in neutrophils links innate immunity to coagulation pathways. J. Immunol..

[29] Risitano A.M. (2013). Paroxysmal nocturnal hemoglobinuria and the complement system: Recent insights and novel anticomplement strategies. Adv. Exp. Med. Biol..

[30] Ekdahl K.N., Fromell K., Mannes M., Grinnemo K.H., Huber-Lang M., Teramura Y., Nilsson B. (2022). Therapeutic regulation of complement activation in extracorporeal circuits and intravascular treatments with special reference to the alternative pathway amplification loop. Immunol. Rev..

[31] Stahl G.L., Shernan S.K., Smith P.K., Levy J.H. (2012). Complement activation and cardiac surgery: A novel target for improving outcomes. Anesth. Analg..

[32] Lazar H.L., Bokesch P.M., van Lenta F., Fitzgerald C., Emmett C., Marsh H.C., Ryan U., OBE and the TP10 Cardiac Surgery Study Group (2004). Soluble human complement receptor 1 limits ischemic damage in cardiac surgery patients at high risk requiring cardiopulmonary bypass. Circulation.

[33] Shernan S.K., Fitch J.C., Nussmeier N.A., Chen J.C., Rollins S.A., Mojcik C.F., Malloy K.J., Todaro T.G., Filloon T., Boyce S.W. (2004). Impact of pexelizumab, an anti-C5 complement antibody, on total mortality and adverse cardiovascular outcomes in cardiac surgical patients undergoing cardiopulmonary bypass. Ann. Thorac. Surg..

[34] Mastellos D.C., Ricklin D., Lambris J.D. (2019). Clinical promise of next-generation complement therapeutics. Nat. Rev. Drug Discov..

[35] Aukrust P., Gullestad L., Lappegard K.T., Ueland T., Aass H., Wikeby L., Simonsen S., Froland S.S., Mollnes T.E. (2001). Complement activation in patients with congestive heart failure: Effect of high-dose intravenous immunoglobulin treatment. Circulation.

[36] Kuehn B.M. (2021). FDA: Stop Using Medtronic’s Heartware Ventricular Assist Device. JAMA.

[37] Cho S.M., Mehaffey J.H., Meyers S.L., Cantor R.S., Starling R.C., Kirklin J.K., Jacobs J.P., Kern J., Uchino K., Yarboro L.T. (2021). Cerebrovascular Events in Patients with Centrifugal-Flow Left Ventricular Assist Devices: Propensity Score-Matched Analysis From the Intermacs Registry. Circulation.

[38] Strueber M., O’Driscoll G., Jansz P., Khaghani A., Levy W.C., Wieselthaler G.M., HeartWare I. (2011). Multicenter evaluation of an intrapericardial left ventricular assist system. J. Am. Coll. Cardiol..

[39] Brandwijk R., Michels M., van Rossum M., de Nooijer A.H., Nilsson P.H., de Bruin W.C.C., Toonen E.J.M. (2022). Pitfalls in complement analysis: A systematic literature review of assessing complement activation. Front. Immunol..

[40] Bergseth G., Ludviksen J.K., Kirschfink M., Giclas P.C., Nilsson B., Mollnes T.E. (2013). An international serum standard for application in assays to detect human complement activation products. Mol. Immunol..

