# Supplementary figures and images for: Implantation of a Continuous-Flow Left Ventricular Assist Device During Cardiopulmonary Bypass Is Associated with a Significant and Transient Acute Thromboinflammatory Response
Source: Int J Mol Sci. 2026 May 20;27(10):4594. doi: 10.3390/ijms27104594 (PMC13206958; doi:10.3390/ijms27104594)

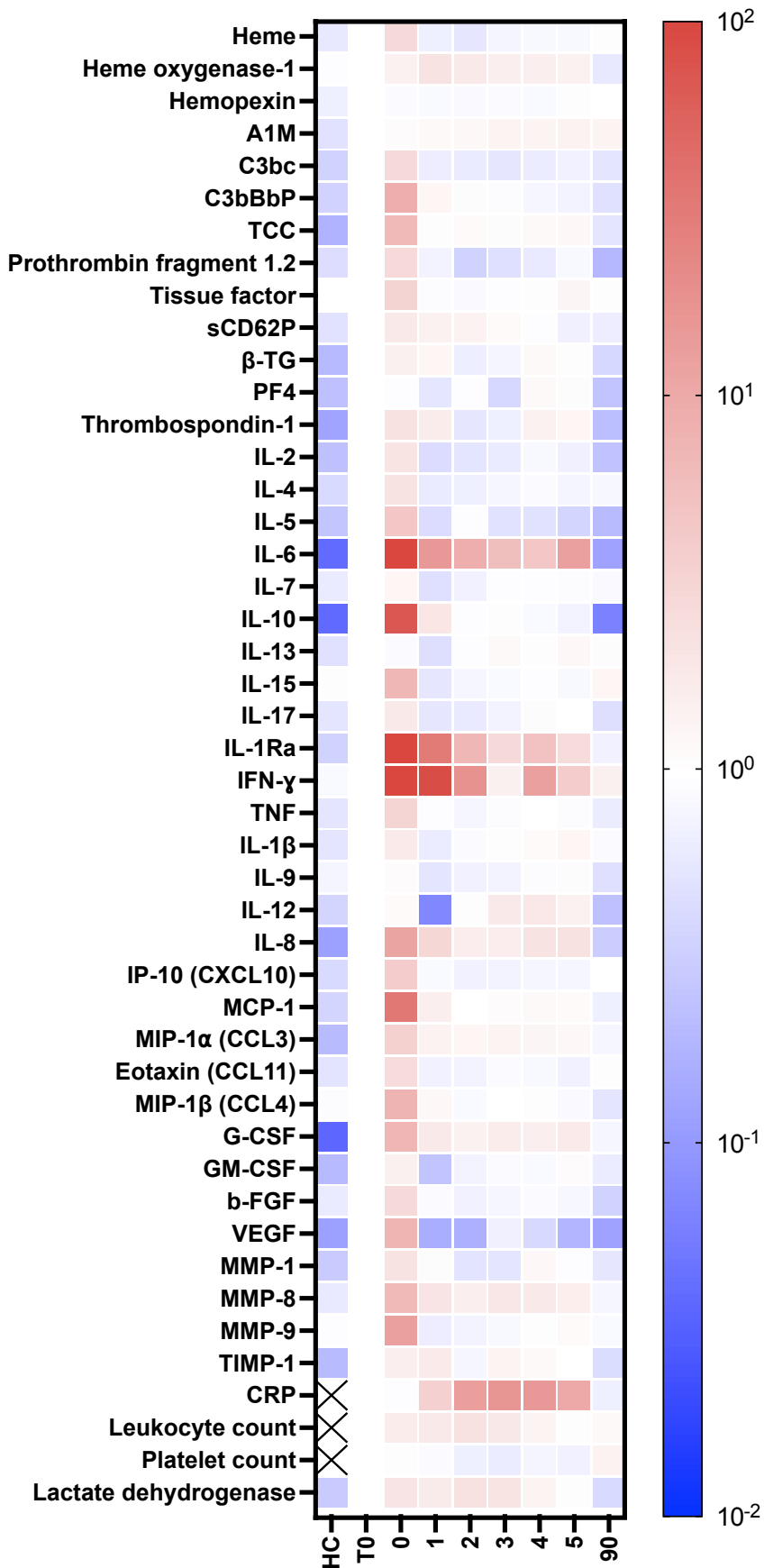

Supplement: Supplementary file 1 [file ijms-27-04594-s001.zip › Supplementary Figure S1.pdf]
